# New adipokines vaspin and omentin. Circulating levels and gene expression in adipose tissue from morbidly obese women

**DOI:** 10.1186/1471-2350-12-60

**Published:** 2011-04-28

**Authors:** Teresa Auguet, Yunuen Quintero, David Riesco, Beatriz Morancho, Ximena Terra, Anna Crescenti, Montserrat Broch, Carmen Aguilar, Montserrat Olona, José Antonio Porras, Mercè Hernandez, Fátima Sabench, Daniel del Castillo, Cristóbal Richart

**Affiliations:** 1 Servei Medicina Interna. Hospital Universitari Joan XXIII Tarragona, Tarragona, Spain; 2 Grup de Recerca en Medicina Aplicada Hospital Joan XXIII. Departament de Medicina i Cirurgia. Universitat Rovira i Virgili (URV), IISPV, Tarragona, Spain; 3 Grup d'estudi de Malalties Metabòliques associades a insulin resistència (GEMMAIR) 2010PFR-URV-B2-14 and 2009 SGR 95 (AGAUR; 4 Servei Epidemiologia i estadística, Hospital Universitari Joan XXIII Tarrogona, Tarragona, Spain; 5 Servei de Cirurgia. Hospital Sant Joan de Reus, Tarragona, Spain. Departament de Medicina i Cirurgia. Universitat Rovira i Virgili (URV), IISPV, Tarragona, Spain

**Keywords:** circulating levels, morbid obesity, mRNA tissue expression, omentin, vaspin

## Abstract

**Background:**

Vaspin and omentin are recently described molecules that belong to the adipokine family and seem to be related to metabolic risk factors. The objectives of this study were twofold: to evaluate vaspin and omentin circulating levels and mRNA expression in subcutaneous and visceral adipose tissues in non-diabetic morbidly obese women; and to assess the relationship of vaspin and omentin with anthropometric and metabolic parameters, and other adipo/cytokines.

**Design:**

We analysed vaspin and omentin circulating levels in 71 women of European descent (40 morbidly obese [BMI ≥ 40 kg/m^2^] and 31 lean [BMI ≤ 25]). We assessed vaspin and omentin gene expression in paired samples of visceral and subcutaneous abdominal adipose tissue from 46 women: 40 morbidly obese and 6 lean. We determined serum vaspin and plasma omentin levels with an Enzyme-Linked Immunosorbent Assay and adipose tissue mRNA expression by real time RT-PCR.

**Results:**

Serum vaspin levels in the morbidly obese were not significantly different from those in controls. They correlated inversely with levels of lipocalin 2 and interleukin 6. Vaspin mRNA expression was significantly higher in the morbidly obese, in both subcutaneous and visceral adipose tissue.

Plasma omentin levels were significantly lower in the morbidly obese and they correlated inversely with glucidic metabolism parameters. Omentin circulating levels, then, correlated inversely with the metabolic syndrome (MS). Omentin expression in visceral adipose tissue was significantly lower in morbidly obese women than in controls.

**Conclusions:**

The present study indicates that vaspin may have a compensatory role in the underlying inflammation of obesity. Decreased omentin circulating levels have a close association with MS in morbidly obese women.

## Background

The incidence of obesity is rising rapidly in industrialized and developing countries. Increased abdominal visceral fat is associated with insulin resistance, type 2 diabetes, and coronary heart disease [1]. Reduction of visceral fat mass by omentectomy has significant positive and long-term effects on glucose metabolism, insulin sensitivity, and metabolic profiles in obese subjects [2]. In the last decade, numerous studies have revealed that adipose tissue secretes a variety of bioactive substances that could explain the epidemiologic relationship between visceral fat mass and increased metabolic risk. These substances, termed adipokines, include leptin [3], adiponectin [4], resistin [5], lipocalin 2(LCN2) [6], fatty acid binding proteins (AFABP) [7], plasminogen activator inhibitor-1 [8], interleukin 6 (IL 6) [9], and various growth factors. They are considered to play an important role in interactions between a variety of systems, including adrenal, immune, and central and peripheral nervous systems [10]. In obese subjects, expression, synthesis and release of pro-inflammatory cytokines (tumor necrosis factor-alpha [TNF-α], IL 6) and adipokines (leptin, resistin) is enhanced but anti-inflammatory adipokines such as adiponectin are decreased [11].

Recently, vaspin (visceral adipose tissue-derived serine protease inhibitor) was identified as a member of the serine protease inhibitor family. Vaspin cDNA was isolated from visceral white adipose tissues (WAT) of Otsuka Long-Evans Tokushima Fatty (OLETF) rats, an animal model of abdominal obesity with type 2 diabetes [12]. Vaspin is highly expressed in rat adipocytes from visceral WAT at the age when obesity and insulin plasma concentrations reach a peak [13]. Vaspin improves insulin sensitivity in mice [13]. However, in humans the effect of vaspin on insulin sensitivity is uncertain and the correlation between vaspin and body mass index (BMI) is also unclear [14,15].

Omentin 1 was identified as a novel adipokine predominantly secreted by visceral stromal vascular cells but not adipocytes [16,17]. Furthermore, in vitro experiments revealed that treatment with recombinant omentin-1 enhances insulin-mediated glucose uptake in human subcutaneous and omental adipocytes, while increasing Akt/PKB phosphorylation [17]. In cultured adipocytes, omentin 1 production is decreased by D-glucose and insulin [18,19].

Nevertheless, in studies involving humans, the plasma concentration of omentin-1-the major circulating isoform in human plasma-is decreased in patients with type 1 diabetes mellitus [18,19] and not affected by glucose ingestion [20]. In addition, omentin plasma levels and omentin gene expression in visceral adipose tissue are decreased in obesity [19].

Although some studies have focused on the abnormal levels of vaspin and omentin in metabolic syndrome patients and mRNA expression, particularly with reference to BMI and markers of insulin sensitivity, the regulation of these molecules and their relationship with other adipokines in morbidly obese patients has not been specifically studied.

Our objective in this study was to measure the circulating levels of vaspin and omentin and mRNA expression in subcutaneous and visceral adipose tissue in morbidly obese women and make a comparison with age-matched control women (we studied only women to avoid sex differences). We also assessed the relationship between these two adipokines and biochemical markers of metabolic syndrome, levels of other adipokines, and pro-inflammatory cytokines.

## Methods

### Subjects

The study was approved by the institutional review board. All participants gave written informed consent for participation in medical research. We analyzed the circulating levels of vaspin and omentin in 71 Spanish women of European descent: 31 lean (BMI < 25 Kg/m2) and 40 morbidly obese (BMI > 40). We also analyzed vaspin and omentin gene expression in paired samples of subcutaneous and visceral adipose tissue from 46 patients: 6 lean (BMI < 25 Kg/m2) and 40 morbidly obese (BM I > 40). Adipose tissue samples were obtained from morbidly obese women and from control women who underwent bariatric surgery by laparoscopic gastric by-pass and elective surgery, respectively. Subcutaneous adipose tissue biopsies were taken from the right hypocondrion region and visceral adipose tissue biopsies were taken from the greater epiploon region. For each type of surgery, samples were obtained by the same specialist. Morbidly obese women and controls were age matched. The weight of all subjects was stable for at least three months before surgery. Those patients who had an acute illness, acute or chronic inflammatory or infective diseases, or an end-stage malignant disease, were excluded from. Liver and renal diseases were specifically excluded by biochemical work-up. Control or morbidly obese patients diagnosed as type 2 diabetes mellitus or receiving hypolipemiant treatment were also excluded from the study.

### Anthropometric Measurements

BMI was calculated as weight divided by height squared (kg/m^2^) (according to the criteria of the World Health Organization [21]. Waist circumference (WC) was measured at the height of the iliac crest.

### Diagnosis of Metabolic Syndrome

Morbidly obese women were further subclassified according to the presence or absence of the metabolic syndrome (MS). The MS and metabolic risks are defined according to the US National Cholesterol Education Program Adult Treatment Panel III guidelines and modified as recommended in the latest American Heart Association/National Heart, Lung, and Blood Institute Scientific Statement [22] by adopting a lower cutoff for fasting glucose (5.6 mmol/L). The MS was defined as having 3 of the following metabolic risk factors: (1) central obesity (waist circumference 88 cm in women), (2) hypertriglyceridemia (fasting triglycerides 1.69 mmol/L (150 mg/dL)), (3) low HDL cholesterol (fasting HDL <1.29 mmol/L (50 mg/dl) in women), (4) glucose intolerance (fasting glucose 5.6 mmol/L (100 mg/dL)), and (5) hypertension (sitting blood pressure 130/85 mm Hg obtained as a mean of two readings taken after resting for at least 10 minutes or on regular antihypertensive medications).

### Analytical Methods

Basal, fasting blood samples were taken after an overnight fast to determine glucose, insulin, and standard laboratory parameters. Plasma and serum samples were stored at -80°C until analytical measurements were performed, except for glucose, which was determined immediately after blood was drawn.

Fasting plasma glucose and lipid profile (triglycerides, total cholesterol, and high-density lipoprotein cholesterol) were measured using the usual enzymatic methods in an ADVIA Centaur auto analyzer. Low-density lipoprotein cholesterol was calculated as the difference between total cholesterol, HDL, and triglyceride content/5 if it was <400 mg/dL. Plasma insulin concentration was measured by commercial chemiluminescence assay for ADVIA Centaur (Siemens Medical Solutions S.L., Barcelona, Spain) according to the manufacturer's instructions. The homeostasis model assessment of insulin resistance (HOMA2-IR) was completed using the HOMA Calculator version 2.2.2 (http://www.dtu.ox.ac.uk accessed May 2010). Glycosylated hemoglobin (HbA1c) was measured by a chromatographic method (Glico Hb Quick Column Procedure, Helena Laboratories, Beaumont, TX).

Circulating levels of TNF-RI, TNF-RII (Biosource Europe S.A., Nivelles, Belgium), IL 6 (Quantikine, R&D Systems, Minneapolis, USA), adiponectin, HMW adiponectin (Linco Research, Inc., St. Charles, USA), resistin (Biovendor, Modrice, Czech Republic), leptin (Biovendor, Modrice, Czech Republic), RBP4, lipocalin 2 (Biovendor, Modrice, Czech Republic), serum vaspin (Adipogen, Seoul, South Korea) and plasma omentin (Apotech, Axxora, Nottingham, UK) were measured in duplicate using enzyme-linked immunosorbent assays (ELISA) following the manufacturer's instructions. TNF-RI assay sensitivity was 50 pg/mL and the inter-assay and intra-assay coefficients of variation were less than 5.7 and 1.7%, respectively. TNF-RII assay sensitivity was 0.1 ng/mL and inter-assay and intra-assay coefficients of variation were less than 3.2 and 3.3%, respectively. IL 6 assay sensitivity was 0.039 pg/mL and inter-assay and intra-assay coefficients of variation were less than 9.6 and 6.9%, respectively. Adiponectin assay sensitivity was 0.78 ng/mL and inter-assay and intra-assay coefficients of variation were less than 8.4 and 7.4%, respectively. HMW adiponectin assay sensitivity was 0.5 ng/mL and inter-assay and intra-assay coefficients of variation were less than 3.8 and 2.6%, respectively. Resistin assay sensitivity was 33 pg/mL and inter-assay and intra-assay coefficients of variation were less than 6.9 and 3.4%, respectively. Leptin assay sensitivity was 0.2 ng/mL and inter-assay and intra-assay coefficients of variation were less than 7.6 and 4.4%, respectively. RBP4 assay sensitivity was 0.02 g/L and inter-assay and intra-assay coefficients of variation were less than 1.1 and 2.2%, respectively. LCN 2 assay sensitivity was 0.01 ng/mL and inter-assay and intra-assay coefficients of variation were less than 5.6 and 4.4%, respectively. Vaspin assay sensitivity was 12 pg/mL and inter-assay and intra-assay coefficients of variation were <6% and <4%. Omentin assay sensitivity was 0.4 ng/mL and inter-assay and intra-assay coefficients of variation were less than 6 and 2.7%, respectively.

### Analysis of Human Vaspin and Omentin Gene Expression

Total RNA was isolated from adipose tissues with the RNeasy mini kit (Qiagen) according to the manufacturer's protocol and digested with DNase I (RNase-Free DNase set, Qiagen). RNA quality was evaluated by measuring the 260/280 nm absorbance ratio (≥1.8) and by electrophoresis. First-strand cDNA was synthesized using an equal amount of total RNA with the High Capacity RNA-to-cDNA Kit (Applied Biosystems). Real-time quantitative PCR was performed in a final volume of 20 μL, which contained 10 ng of reverse-transcribed cDNA, 10 μL of 2X Taq Man Fast Universal PCR Master Mix (Applied Biosystems) and 1 μL Taq Man Assay predesigned by Applied Biosystems^® ^for the detection of vaspin, omentin, and GAPDH, used as a housekeeping gene. All reactions were performed in triplicate and were carried out in 96-well plates by using the 7900HT Fast Real-Time PCR systems (Applied Biosystems).

### Statistical Analysis

All the values reported are expressed as mean ± SEM (standard error of the mean) and were analyzed using the statistical package SPSS/PC+ for Windows (v.15.0 Chicago, Illinois, USA). Differences between groups were calculated using either Student's t test or the One-way ANOVA analysis. The strength of association between variables was calculated using Pearson's method for parametric variables and the Spearman Rho correlation test for non-parametric contrasts. Multiple linear regression analysis with backward variable selection was performed to identify independent predictors of HOMA2-IR. The validity of the regression model and its assumptions was assessed with the plot of residuals vs. predicted values. Data were normally distributed. Logistic regression analysis was performed to identify independent predictors of the metabolic syndrome. Vaspin and omentin circulating levels were age and BMI adjusted in some analyses. P values < 0.05 were considered to be statistically significant.

## Results

### Population studied

The baseline patient characteristics given in Table 1 show the mean and SEM of the variables of interest. Patients were separated into control subjects (BMI < 25 kg/m^2^), and morbid subjects (BMI > 40 kg/m^2^). The two groups were well matched for age.

**Table 1:** Baseline characteristics, anthropometric measurements, and metabolic analysis of the population studied

	CONTROL (n = 31)	MORBID OBESE (n = 40)	*p*-value
**BMI (Kg/m**^**2**^**)**	23.21 ± 0.46	48.22 ± 1.07	**<0.001**
**WC (cm)**	80.40 ± 2.16	134.00 ± 3.10	**<0.001**
**AGE (years)**	43.53 ± 3.08	46.37 ± 1.54	0.414
**GLUCOSE (mg/dL)**	94.06 ± 2.53	117.70 ± 4.25	**0.001**
**INSULIN (mU/L)**	8.25 ± 1.32	22.69 ± 3.94	**0.001**
**HOMA2-IR**	1.11 ± 0.18	2.60 ± 0.24	**<0.001**
**HbA1c (%)**	4.55 ± 0.07	5.63 ± 0.29	**0.001**
**HDL-C (mg/dL)**	59.83 ± 2.87	40.97 ± 1.33	**<0.001**
**TRIGLYCERIDES (mg/dL)**	95.13 ± 9.06	183.52 ± 11.88	**<0.001**
**SBP (mm Hg)**	120.91 ± 4.22	134.99 ± 4.07	**0.020**
**DBP (mm Hg)**	70.04 ± 1.96	76.70 ± 2.80	0.056

BMI: body mass index, WC: waist circumference, HOMA2-IR: homeostatic model assessment method insulin resistance, HDL-C: high density lipoprotein cholesterol, SBP: systolic blood pressure, DBP: diastolic blood pressure. Data are the mean ± SEM. * indicates significant differences vs. control group (p < 0.05).

Biochemical analyses indicated that obese women had significantly higher levels of glucose, insulin, HOMA2-IR and HbA1c than the control group. Blood pressure was also increased in the morbidly obese women. The lipidemic profile differed significantly between groups. Obese patients showed higher triglyceride levels and lower HDL cholesterol.

### Serum vaspin

**Figure 1:**
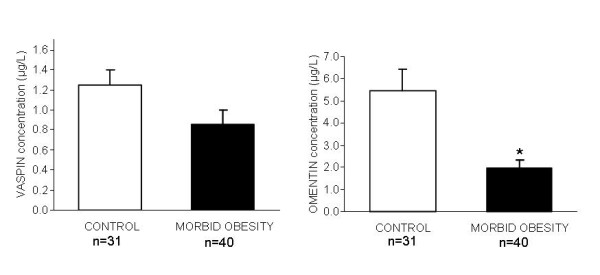
**Vaspin and omentin concentrations in control women and morbidly obese women**. * Indicates statistically significant differences between groups (p < 0.05).

Mean ± SD serum vaspin was 0.87 ± 0.96 μg/liter in the morbidly obese women and not significantly different from the values of the control group (1.66 ± 2.09 μg/liter) (Figure 1).

Serum vaspin concentrations did not correlate with metabolic variables (Table 2) and inversely correlated with levels of LCN2, leptin and IL 6 (Table 3).

**Table 2:** Correlation of vaspin and omentin levels with metabolic variables

Circulating levels	VASPIN		OMENTIN	
**Variables**	**r**	***p*-value**	**r**	***p*-value**

**BMI (kg/m**^**2**^**)**	-0.181	0.134	-0.212	0.096
**WC (cm)**	-0.448	**0.007**	-0.332	0.078
**Glucose (mg/dL)**	-0.002	0.989	-0.276	**0.029**
**Insulin (mU/L)**	-0.015	0.903	-0.252	0.054
**HOMA2-IR**	0.004	0.976	-0.274	**0.037**
**HbA1c (%)**	-0.181	0.218	0.011	0.943
**HDL-C (mg/dL)**	0.166	0.183	-0.178	0.182
**Triglycerides (mg/dL)**	-0.218	0.072	-0.101	0.433
**SBP (mm Hg)**	-0.002	0.986	-0.179	0.201
**DBP (mm Hg)**	-0.019	0.887	-0.072	0.608

BMI: body mass index, WC: waist circumference, HOMA2-IR, homeostatic model assessment method insulin resistance; HDL-C: high density lipoprotein cholesterol, SBP: systolic blood pressure, DBP: diastolic blood pressure. Bolded p-values indicate statistically significant correlations (p < 0.05).

**Table 3:** Correlation of vaspin and omentin levels with circulating adipo/cytokine levels

Circulating levels	VASPIN		OMENTIN	
**Variables**	**r**	***p*-value**	**r**	***p*-value**

**HMW adiponectin (μg/L)**	0.178	0.173	0.078	0.557
**Adiponectin (μg/L)**	0.145	0.250	0.103	0.431
**Resistin (μg/L)**	-0.178	0.157	-0.150	0.236
**LCN2 (μg/L)**	-0.336	**0.016**	0.088	0.559
**IL6 (ng/L)**	-0.290	**0.029**	0.033	0.812
**RBP4 (mg/dL)**	0.015	0.915	0.013	0.931
**TNFRI (μg/L)**	-0.241	0.057	0.004	0.976
**TNFRII (μg/L)**	-0.051	0.689	0.036	0.787
**Leptin (μg/L)**	-0.290	**0.041**	0.195	0.199

HMW adiponectin: high molecular weight adiponectin, LCN2: lipocalin 2, IL6: interleukin 6, RBP4: retinol binding protein 4, TNFRI: tumor necrosis factor receptor I, TNFRII: tumor necrosis factor receptor II. Bolded p-values indicate statistically significant correlations (p < 0.05).

We investigated the relationship between vaspin circulating levels and the presence of the MS but we found no correlation (Table 4). As expected, the MS correlated with the BMI, WC, HOMA2-IR, fasting glucose, blood pressure, HDL cholesterol and triglycerides.

**Table 4:** Correlations between the number of diagnostic criteria of metabolic syndrome met by the patients

	Number of criteria of Metabolic syndrome	
	
Variables	r	*p*-value
**VASPIN (μg/L)**	-0.209	0.083
**OMENTIN (μg/L)**	-0.264	**0.045**
**BMI (kg/m**^**2**^**)**	0.737	**<0.0001**
**WC (cm)**	0.783	**<0.0001**
**HOMA2-IR**	0.589	**<0.0001**
**Glucose (mg/dL)**	0.710	**<0.0001**
**SBP (mm Hg)**	0.636	**<0.0001**
**DBP (mm Hg)**	0.250	**0.006**
**HDL-C (mg/dL)**	-0.750	**<0.0001**
**Triglycerides (mg/dL)**	0.704	**<0.0001**

Vaspin and omentin circulating levels and different parameters.

BMI: body mass index, WC: waist circumference, HOMA2-IR: homeostatic model assessment method insulin resistance, SBP: systolic blood pressure, DBP: diastolic blood pressure.

Bolded p-values indicate statistically significant Spearman's correlations (p < 0.05).

### Subcutaneous and visceral vaspin mRNA expression

**Figure 2:**
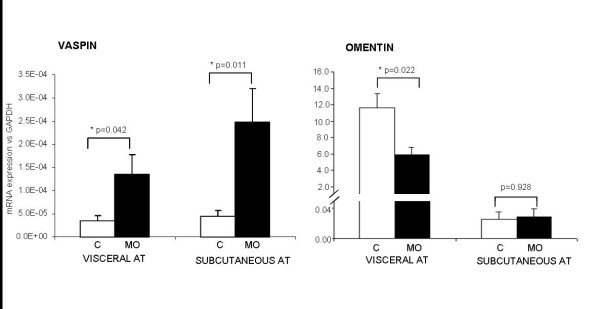
**mRNA vaspin and omentin expression in control group (C) (n = 6) and morbidly obese women (MO) (n = 40)**. * indicates statistically significant differences between control and morbidly obese subjects (p < 0.05).

Vaspin mRNA expression was significantly higher in morbidly obese women than in controls, in both SAT and VAT (Figure 2). We found no differences between visceral and subcutaneous vaspin expression in the adipose tissues of the obese group (p = 0.192) and the control group (p = 0.934) (Figure 2).

SAT and VAT vaspin mRNA expression did not correlate with obesity, glucose metabolism, insulin resistance parameters, blood pressure, other adipokines studied or inflammatory parameters (data not shown).

### Plasma omentin-1

Mean ± SD plasma omentin was 1.97 ± 2.15 ng/mL in the morbidly obese group and significantly different from that of the control group (5.27 ± 5.33 ng/mL) (Figure 1).

Plasma omentin levels inversely correlated with fasting glucose and HOMA2-IR (Table 2) and did not correlate with the adipokines or cytokines studied (Table 3). When we investigated the relationship between omentin circulating levels and the presence of the MS, we found that omentin correlated negatively with the MS (Table 4). After adjustment for age and BMI, we subclassified the subjects in three tertiles in accordance with their omentin levels: tertile 1 (>4.33 ng/ml); tertile 2 (4.33-2.30 ng/ml); tertile 3 (<2.30 ng/ml). In a logistic regression analysis, the lowest omentin levels (tertile 2 and 3) were associated with the presence of MS while the highest tertile (tertile 1) was not (Table 5).

**Table 5:** Logistic regression analysis for the presence of the metabolic syndrome according to the omentin tertile circulating levels

	OR	95% CI	p-value
**OMENTIN tertile 2 vs 1**	25.00	4.41-141.68	**<0.001**
**OMENTIN tertile 3 vs 1**	90.00	11.46-706.71	**<0.001**

Model 1. Omentin tertiles (ng/ml) adjusted for BMI and age: 1 (>4.33); 2 (4.33-2.30); 3 (<2.30). Tertile 1 as reference with OR = 1.

### Visceral and subcutaneous omentin expression

Omentin mRNA expression in SAT was similar in morbidly obese women and controls. However, omentin expression in VAT was significantly lower in morbidly obese women than in controls (Figure 2). When comparing both adipose tissues, we found that omentin expression was higher in VAT than in SAT in both the obese group (p = 0.009) and the control group (p = 0.034) (Figure 2).

SAT and VAT omentin mRNA expression did not correlate with obesity, glucose metabolism or insulin resistance parameters. Neither was there any correlation with lipid metabolism, blood pressure or the other adipokines and cytokines studied (data not shown).

## Discussion

It has been reported that abnormalities in the circulating levels of vaspin and omentin and the gene expression of both factors are related to BMI and markers of insulin sensitivity in metabolic syndrome patients, although to date, the findings of various authors are confusing. Our objective in this study was to measure vaspin and omentin circulating levels and mRNA expression in subcutaneous and visceral adipose tissue in morbidly obese women and to compare them to age-matched controls. We also assessed the relationship between these two adipokines and biochemical markers of metabolic syndrome and other adipo/cytokines.

In the current study, we demonstrate that serum vaspin levels are not increased in morbidly obese women and that vaspin levels do not correlate with BMI, markers of glucose or lipid metabolism.

In studies involving humans, and as has been mentioned in the introduction, how vaspin serum levels correlate with BMI, markers of insulin sensitivity and glucose metabolism is unclear. Youn et al. have reported that elevated vaspin serum concentrations correlated with obesity and impaired insulin sensitivity, although not in patients with type 2 diabetes [14]. In obese children, Lee et al. have observed a negative correlation between vaspin concentration and HOMA-IR [23]. However, in agreement with our results, von Loeffelholz et al. have shown that there is no association between serum vaspin and HOMA-IR in nondiabetic humans [24]. Seeger et al. have also reported that circulating vaspin is not independently associated with markers of glucose metabolism [15], and Briana et al. have shown that vaspin concentrations do not correlate with insulin levels in maternal, fetal and neonatal samples [25], as occurs in our population.

To our knowledge, this is the first time that the relation between vaspin levels and other adipo/cytokines in the circulation has been studied.

In our study, serum vaspin levels correlate inversely with levels of LCN2 and IL 6. It has been reported that LCN2 is an adipokine that seems to be an independent risk factor for hyperglycemia and insulin resistance in humans. It has also been related to inflammation [26]. IL 6 is a known proinflammatory cytokine that also increased in obesity [9]. Taken together, these findings might suggest that vaspin has an anti-inflammatory profile.

On the other hand, vaspin mRNA expression is significantly higher in our morbidly obese cohort in SAT and VAT. Kloting et al. have reported that vaspin expression was not detectable in lean subjects but that it was present in both the SAT and VAT of obese patients. Its levels were significantly correlated with parameters of obesity, insulin resistance and impaired glucose tolerance [27]. However, we have not been able to support their findings.

In untreated OLETF rats, vaspin expression and its serum levels decreased as diabetes worsened and body weight fell. The expression and serum levels were normalized by treatment with insulin or pioglitazone, suggesting that vaspin exerts a defensive action against insulin resistance. On the other hand, the administration of recombinant human vaspin improved insulin sensitivity and glucose tolerance, and reverses the expression of those genes that can promote insulin resistance such as leptin, resistin and TNF-α, in diet-induced obese mice [13].

To sum up, in our study serum vaspin levels are inversely related to IL 6. SAT and VAT vaspin expression is significantly higher in morbidly obese women. In addition, as mentioned above, the literature confirms that vaspin has an insulin-sensitizing effect. In conjunction with our results, then, this suggests that vaspin has a compensatory role in the inflammatory complications of obesity.

The second important finding of our study is that plasma omentin levels are significantly lower in the morbidly obese and that these levels inversely correlate with glucidic metabolism parameters, in accordance with the findings of de Souza et al. [19] and Yang et al. [17]. In the same context, we found a negative correlation with systolic blood pressure.

We also demonstrate that patients with omentin levels in the lowest tertile were 90 times more likely to have the MS than those in the highest tertile, after adjustment for age and BMI. Moreover, women with omentin levels in the second tertile were 25 times more likely to have the MS.

Omentin expression in visceral adipose tissue is significantly lower in the morbidly obese women in our study in agreement with the results of Souza et al [19]. Also, Cai et al. demonstrate that omentin mRNA expression decreases in overweight/obese individuals and decreases further when overweight/obesity is combined with type 2 diabetes. Thus, omentin expression is negatively correlated with fasting insulin, HOMA-IR and BMI [28].

The major limitation of the present study is the relatively small number of subjects in the sample. Although our specific cohort of non-diabetic morbidly obese women showed a clear relationship between the MS and omentin levels without the interference of confounding factors, these results are not extrapolable to other obesity groups or men. Secondly, due to the difficulty of obtaining tissue samples, the expression results need to be confirmed in larger study populations. Another limitation of the study is that it is cross-sectional. We could not prove a causal link between the levels of omentin and the development of MS or the levels of vaspin and anti-inflammatory action. Further prospective studies are required to explain these phenomena.

## Conclusion

To sum up, our data suggest that vaspin is likely to have anti-inflammatory/protective action in morbid obesity. Also, decreased omentin levels have a close association with MS in women with morbid obesity. Future studies should also include parameters for weight loss and exercise to observe their effect on vaspin and omentin levels.

## Abbreviations

BMI: body mass index; DBP: diastolic blood pressure, HOMA2-IR: homeostatic model assessment method insulin resistance; HMW: adiponectin, high molecular weight adiponectin; IL6: interleukin 6; LCN2: lipocalin 2; MS: metabolic syndrome; RBP4: retinol binding protein 4; SAT: subcutaneous adipose tissue; SBP: systolic blood pressure; TNF-α: tumor necrosis factor-alpha, TNF-RI: tumor necrosis factor receptor I; TNF-RII: tumor necrosis factor receptor II; VAT: visceral adipose tissue; WC: waist circumference.

## Competing interests

The authors declare that they have no competing interests.

## Authors' contributions

YQ, BM, AC, MB and CA carried out the molecular genetic studies and the immunoassays. TA participated in the design of study, performed the statistical analyses and was involved in drafting the manuscript. XT carried out the molecular genetic studies and performed the statistical analysis. MO performed the statistical analysis. DR, JAP, MH and FS made substantial contributions to the conception and design, acquisition of data, and analysis and interpretation of data. They were also involved in drafting the manuscript. DdC and CR revised the draft and gave final approval for publication. All authors read and approved the manuscript.

## Pre-publication history

The pre-publication history for this paper can be accessed here:

http://www.biomedcentral.com/1471-2350/12/60/prepub
